# Microfluidic Long-Term Gradient Generator with Axon Separation Prototyped by 185 nm Diffused Light Photolithography of SU-8 Photoresist

**DOI:** 10.3390/mi10010009

**Published:** 2018-12-24

**Authors:** Nobuyuki Futai, Makoto Tamura, Tomohisa Ogawa, Masato Tanaka

**Affiliations:** 1 Department of Mechanical Engineering, Shibaura Institute of Technology, 3-7-5 Toyosu, Koto-ku, Tokyo 135-8548, Japan; aa11070@shibaura-it.ac.jp; 2 Department of Human Pathology, Tokyo Medical and Dental University, Bunkyo-ku, Tokyo 113-8510, Japan; ogawpth1@tmd.ac.jp; 3 Division of Life Science, Tokyo Denki University, Hiki-gun, Saitama 350-0394, Japan; mtanaka@mail.dendai.ac.jp

**Keywords:** SU-8, microchannel, prototyping, microfluidic gradient generator, axon elongation

## Abstract

We have developed a cast microfluidic chip for concentration gradient generation that contains a thin (~5 µm^2^ cross-sectional area) microchannel. The diffusion of diffused 185 nm ultraviolet (UV) light from an inexpensive low-pressure mercury lamp exposed a layer of the SU-8 photoresist from the backside and successfully patterned durable 2 µm-high microchannel mold features with smooth bell-shaped sidewalls. The thin channel had appropriate flow resistance and simultaneously satisfied both the rapid introduction of test substance and long-term maintenance of gradients. The average height and width at the half height of the channel, defined by a 2 µm-wide line mask pattern, were 2.00 ± 0.19 µm, and 2.14 ± 0.89 µm, respectively. We were able to maintain the concentration gradient of Alexa Fluor 488 fluorescent dye inside or at the exit of the thin microchannel in an H-shaped microfluidic configuration for at least 48 h. We also demonstrated the cultivation of chick embryo dorsal root ganglion neuronal cells for 96 h, and the directional elongation of axons under a nerve growth factor concentration gradient.

## 1. Introduction

Chemical or biomolecule concentration gradients in the microenvironment play a significant role in cellular behaviors, such as axon guidance, and precise targeting over long distance. Microfluidic systems are promising tools to maintain such concentration gradients for a longer duration than traditional devices due to their size matched to the scale of gradients, high throughput, and ease of integration of downstream assays [1,2]. Microfluidic gradient generators have been applied to other microorganisms such as bacteria [3] and roundworms [4].

However, as described in a review containing a case study of traditional and microfluidic chemotaxis chambers [5], traditional devices and their modification are still adopted more than microfluidic gradient generators. Microfluidic devices, including gradient generators, tend to compromise ease of production and use in exchange for their functionality and technical novelty. The Boyden Chamber, a traditional chemotaxis chamber, was designed and fabricated within the scale and methods of conventional machining, not micro-machining; however, in doing so, a highly reproducible and useful device was made [6]. Therefore, simplicity in microfabrication processes and easy handling are requirements in newly developing microfluidic gradient generators. Therefore, a simplicity in microfabrication processes and easy handling are requirements in newly developing microfluidic gradient generators. In addition, the current microfluidic gradient generation devices have relatively low flow resistance. Moreover, it is easy to change the flow by small differences in pressure at the ends. As a result, an end user requires precise control of microflow or pressure to reproduce a consistent concentration profile in a microchannel.

We have developed a one-layer H-shaped microfluidic channel that consists of a thin (~2 µm in top height) but high-aspect-ratio (0.5–1.0) microchannel. The high flow resistance of the microfluidic channel easily maintains the gradient of test substances applied to the channel end with easy pipetting. The concentration profile can also be easily predicted by considering the end of the thin channel as a point source of diffusion. The design of the chip is also comparable to traditional chemotaxis chambers, such as the Zigmond Chamber [7] or Dunn Chamber [8], allowing for a straightforward understanding of its usage. The microfluidic channel is softlithographically replicated from a channel feature mold and bonded to the inner bottom of a commercially available cell culture dish.

To fabricate the casting mold with small microchannel features, we have also developed a simple fabrication method for a durable microchannel mold with ~5 µm^2^ cross-sectional area that only requires a low pressure mercury lamp, commercially available, as an ozone lamp in addition to the SU-8 photoresist [9]. The simplicity of this method was well fitted to softlithographic rapid prototyping of microfluidic devices using SU-8 and low-resolution emulsion photomasks [10]. An ozone lamp was used to overcome the limitation of this previous rapid prototyping method in resolution of SU-8 features [11] (larger than 8 µm) due to insufficient ultraviolet (UV) light dose to SU-8 through photomasks with insufficient transparency.

Soft lithography-friendly prototyping of features, smaller than 1 µm, has been achieved by using several non-UV patterning methods, such as e-beam direct writing of SU-8 photoresist [12], nanocracking [13] and roof collapse [14]. These non-UV methods are simple, but can be either costly or have limitations in how the channel layout can be defined. We exposed SU-8 photoresists to diffused short-wavelength (185 nm) UV light emitted from an ozone lamp through the aperture of the Cr layer between the SU-8 layer and the glass substrate. An emulsion mask could be used to pattern the Cr layer and could successfully define the size and layout of the thin channels. The usefulness of the resultant microfluidic device and the long-term maintenance of gradient generation were demonstrated by in-channel cultivation of primary neuronal cells from chick embryo and guiding their axons through the thin channel. 

## 2. Materials and Methods

### 2.1. Design of the Microfluidic Chip

**Figure 1:**
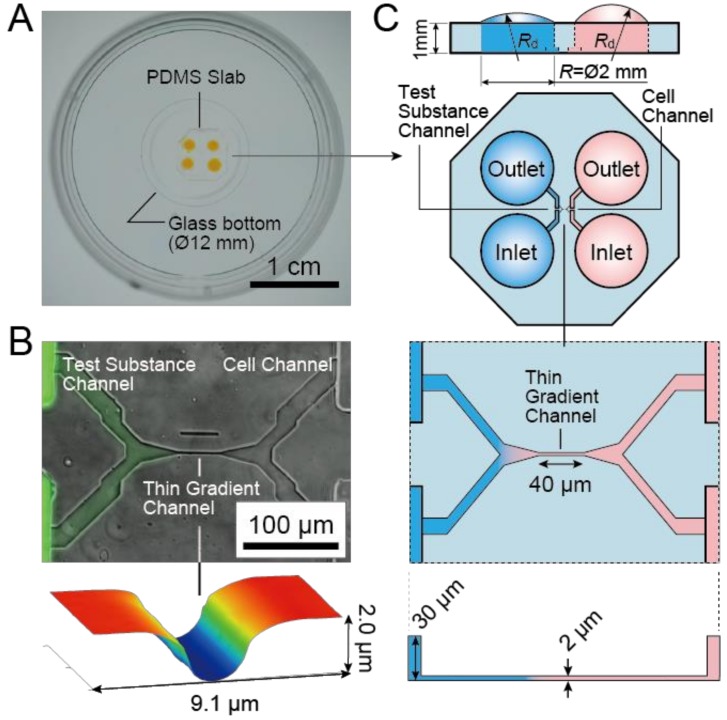
(**A**) A microfluidic gradient generator: a poly(dimethylsiloxane) (PDMS) slab with microchannel features and four holes bonded on a glass-bottom dish. (**B**) Micrograph of the microfluidic channels at the center of the PDMS slab. Fluorescence dye introduced to the channels is overlaid on the phase contrast image. A gradient of fluorescence is generated from the Test Substance Channel to the Cell Channel through the Thin Gradient Channel. A height image of the thin channel indicates that the top height and the width of the thin channel are approximately 2.0 µm and 3 µm. (**C**) Illustration of the channel configuration of the microfluidic chip: a symmetrical H-shaped channel with a thin channel at the center. No cell body but axons can pass through the thin channel. Due to the high flow resistance of the thin channel and slow diffusion rate of the test factor, a concentration gradient along the thin channel is generated.

Figure 1 shows an overview and the basic design of the microfluidic gradient generator chip. As shown in Figure 1A, a poly(dimethylsiloxane) (PDMS) slab with microfluidic channel features with four reservoirs was bonded on a glass-bottom dish. Figure 1B shows the microchannels including one thin channel with bell-shaped sidewalls (2 µm high × 40 µm long) and with bifurcate ends. As shown in Figure 1C, each branch of the bifurcated ends of the thin channel was connected to a thick channel (200 µm wide × 30 µm high). The H-shaped configuration with mixed-height microchannels facilitates the introduction and replacement of liquids in and near to the thin channel.

The H-shaped channel is both horizontally and vertically symmetrical, and either the left or right port pair can be used to introduce cells or test substances. Although in Figure 1C the left channel is assigned to the test substance and the right to cells, the assignment can be switched. Also, the inlet and the outlet can be switched as long as they are on the same side. The left end of the thin channel that connected to a widening channel can be considered as a point source of a test substance. The thick channel at the lower right of the H-shape is used to introduce cells, and the cells stop at the junction to the thin channel. The 30 µm-high channels allow for easy cell loading under low shear stress and allow cell culture within the channels for several days. 

Applying Laplace pressures at the inlet/outlets by placing droplets on them can easily generate flow through the chip. Since the microfluidic route, including the thin segment, has high fluidic resistance, only macroscopic differences between the size of droplets on the inlet and outlet generate flow. This insensitivity makes it easy to start/stop the flow in the thin channel and to generate gradients using a commercially available single channel pipette.

The Laplace pressure P for the droplet height $h_{d}$ is estimated as Equation (1) when the droplet surface over the channel inlet or outlet of radius R can be considered a partial sphere.

(1) $$P=\frac{2\sigma }{R_{d}},\;\;R_{d}=\frac{h}{2}+\frac{R^{2}}{8h}\;,$$

where σ is the surface tension (72.8 × 10^−3^ N/m for water at 20 °C); $R_{d}$ is the geometrically derived radius of the droplet. Since the depth and the diameter of the inlets/outlets are 1 mm and 2 mm, respectively, we can easily generate Laplace pressures ranging from approximately −60 to +60 Pa by adding or subtracting $h_{d}$ from the chip surface level of water with manual pipetting. 

### 2.2. Fabrication of a Microfluidic Chip

**Figure 2:**
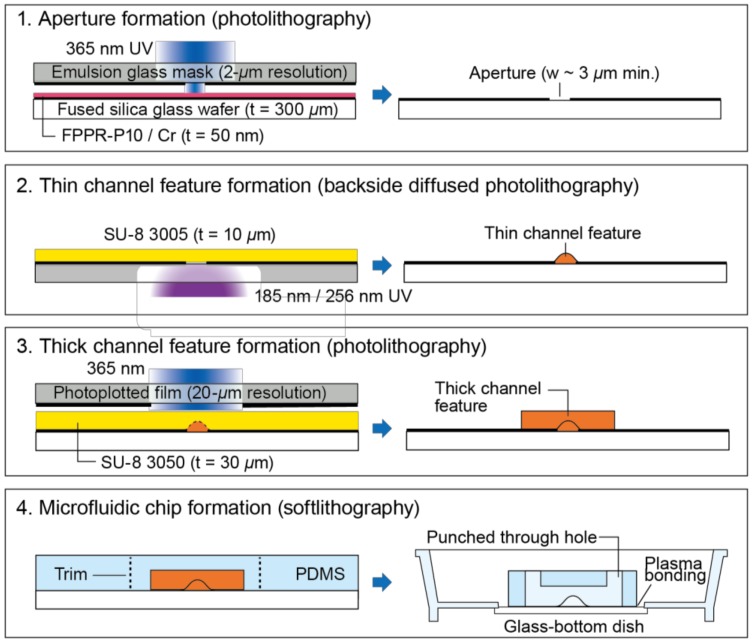
Fabrication of a microfluidic chip with thin and thick mixed-height microchannels. The first three steps illustrate the fabrication of a channel mold, and the remaining steps describe the casting and assembly of the chip. Process overview: (**1**) patterning of positive photoresist onto sputtered Cr film on a fused silica glass wafer, then wet etching of Cr to make the apertures that serve as a photomask, (**2**) patterning of thin channel features (2–20 µm base width) by high-energy ultraviolet (UV) exposure of SU-8 photoresist through the Cr apertures, (**3**) patterning of another SU-8 layer into the thick channel features (~200 µm wide) by conventional photolithography, (**4**) casting of PDMS using the channel features as a mold, and bonding of the cast PDMS onto a glass-bottom dish.

The fabrication processes are illustrated in Figure 2. The photopatterning of SU-8 photoresist was performed twice to successfully define both thin and thick microchannel features. The first patterning of SU-8 photoresist into thin channel features required a high exposure energy dose only into the small regions of the unexposed SU-8 layer. Thus, the combination of backside exposure of SU-8 photoresist [15] and exposure to 185 nm UV was used for the first SU-8 patterning. The photomask for the first exposure must be in contact with the bottom surface of the SU-8 layer to minimize blurring and detachment of SU-8 features from the substrate. Therefore, the SU-8 layer was coated on the patterned Cr film on the glass substrate. The second patterning of SU-8 photoresist consisted of conventional processes using standard 365 nm UV with a glass emulsion photomask.

First, the layout of thin channels defined by apertures on a low-cost emulsion photomask was transferred to a Cr film on a glass wafer. A 50 nm Cr film was deposited on a 300 µm-thick synthetic fused silica glass wafer (50 mm × 50 mm, Seiken Glass, Tokyo, Japan) using a sputter coater (SVC-700RFII, Sanyu Electron, Tokyo, Japan). The wafer was then coated with a 1 µm thick layer of positive photoresist (FPPR-P10, Fuji Chemicals Industrial, Tokyo, Japan) by spin-coating at 5000 rpm for 30 s and then baking at 95 °C for 2 min. The photoresist layer was exposed to collimated 365 nm UV from a spot UV source (L7212-02, Hamamatsu, Shizuoka, Japan) with a dose of 125 mJ/cm^2^ through an emulsion glass photomask (2 µm resolution, Shin-eisha, Tokyo, Japan). The photoresist layer was then developed with 2.38% TMAH (Tama Chemicals, Kanagawa, Japan) and hard baked at 95 °C for 10 min. The exposed Cr film was wet etched in a solution containing 17 wt % (NH_4_)_2_Ce(NO_3_)_6_ and 7 wt % NH_4_NO_3_ (Wako Chemical, Osaka, Japan) in water. The photoresist was removed with dimethyl sulfoxide followed by immersion in 1 vol% alkaline cleaning solution (TMSC, Tama Chemicals, Kanagawa, Japan) at 65 °C for 10 min.

Next, the SU-8 photoresist layer on the patterned Cr film was exposed to UV from the backside to form thin channel features. A negative photoresist (SU-8 3005, Nippon Kayaku, Tokyo, Japan) was spun at 4500 rpm for 30 s to form a layer with a thickness range of 8–10 µm and was dried at 95 °C for 10 min. The SU-8 layer was then exposed to 185 nm and 254 nm UV from a low-pressure mercury vapor lamp (GL-4Z, Kyokko Denki, Tokyo, Japan). The UV power was measured as 0.2 mW/cm^2^ @ 185 nm with a UV power meter (C9536/H9535, Hamamatsu, Japan). The exposure time was controlled by an electronic shutter (DSS10, Uniblitz, Rochester, NY, USA) so that the exposure dose was 0.1 mJ/cm^2^. 

Following the backside exposure, a thick SU-8 layer was patterned using conventional methods. A 30 µm thick negative photoresist layer (SU-8 3050, Nippon Kayaku, Tokyo, Japan) was coated by spinning at 5000 rpm for 30 s, dried, and exposed to the collimated 365 nm UV with a dose of 300 mJ/cm^2^ through an emulsion glass photomask (20 µm resolution, Topic-dic, Tokyo, Japan) aligned to the first SU-8 layer using a 3-axis mechanical stage. Both exposed SU-8 layers were post-exposure baked at 95 °C for 10 min and were developed with 1-methoxy-2-propyl acetate.

Finally, the SU-8 features were transferred to a PDMS slab, which was then bonded to the bottom of a glass bottom dish for cell culture. A PDMS prepolymer (KE106, Shin-etsu, Tokyo, Japan) was poured onto the SU-8-pattened surface to make a 1 mm thick layer, cured at 65 °C for 90 min, and demolded by peeling. A slab containing one H-shaped channel upper was cut from the peeled PDMS, and four 2 mm diameter holes were punched in the circular slab. The punched PDMS slab was bonded to a 35 mm-diameter glass-bottom dish (3971-035, Iwaki, Tokyo, Japan) after air plasma treatment at 20 mA and 40 Pa for 30 s in a plasma chamber (SC-708, Sanyu Electron Co. Ltd., Tokyo, Japan). 

### 2.3. Measurement of Aperture and Channel Dimensions

Cr aperture patterns on the glass wafers were photographed with a laser confocal microscope (OLS4000, Olympus, Tokyo, Japan). The texture and height images of the surface of PDMS slabs with thin channel upper features were acquired by an atomic force microscope (AFM5000II, Hitachi, Tokyo, Japan). Data from both microscopes were analyzed using Gwyddion v2.51 height field and image analysis software [16].

### 2.4. Evaluation of Diffusion on Chip

An assembled chip was primed by introducing 1 µL of phosphate buffered saline (PBS; 10010, Invitrogen, Grand Island, NY, USA) to the inlets/outlets. To avoid bubbles, the water was first poured into one of the inlets, then into its outlet, followed by pouring into the other inlet, and finally into its outlet in series at 15–20 s intervals. To load the thin channel with fluorescence, PBS at the outlet and the inlet of the Test Substance Channel (the left ports shown in Figure 1C) was replaced with 1 µL of 10 µM solution of Alexa Fluor 488 (A33077, Invitrogen, Carlsbad, CA, USA). Next, 1 µL of PBS was added to two ends of Cell Channel; 0.5 µL of PBS and the same Alexa Fluor solution were added to the outlet/inlet of Test Substance Channel. The device was then incubated at 37 °C, 95% RH and 5% CO_2_. A fluorescence laser confocal microscope (FV10i, Olympus, Tokyo, Japan) was used to obtain fluorescence images of the microfluidic channels.

Fluorescence profiles inside the thin channel were fit to the model of one-dimensional static advection-diffusion from a continuous point source [17]:

(2) $$C\left(x\right)=\frac{C_{\max}-C_{\min}}{\exp\mathrm{Pe}-1}\left\{\exp\left(\frac{\mathrm{Pe}\cdot x}{L}\right)-1\right\}+C_{\min}$$

where C(x) is the measured concentration as a function of the position x along the thin channel length L (0≤x≤L), $C_{\max}$ and $C_{\min}$ are the maximum and minimum concentration values within the channel region, and Pe Peclet number is defined using L as the relevant length scale. C(x), $C_{\max}$ and $C_{\min}$ relate to corresponding fluorescence intensity according to Lambert–Beer Law when the fluorescent dye concentration is low enough. We calculated Pe by fitting each fluorescence intensity profile to Equation (2) using Igor Pro 7.0 data analysis software (Wavemetrics, Portland, OR, USA). 

### 2.5. Primary Neural Cell Culture on Chip

For the axon elongation experiment, primary dorsal root ganglion (DRG) cells were introduced to the chip. The DRG cells were freshly isolated from E7 chick embryos by dissection, and were dissociated into DRG cells using 0.25% trypsin (15090, Invitrogen). The channels were coated with a solution of 1 wt % poly-D-lysine (P7886, Sigma, St Louis, MO, USA) and filled with DMEM/F12 medium (11320, Invitrogen) supplemented with ITS solution (#1074547, Roche, Basel, Switzerland).

The inlet and outlet of the Cell Channel were filled with 5 µL of cell suspension containing DRG cells and the medium, respectively. The inlet/outlet of the Test Substance Channel were filled with 5 µL of medium containing 50 ng/mL nerve growth factor (NGF), and the device was then incubated at 37 °C, 95% RH and 5% CO_2_. The cells were observed on an inverted phase contrast microscope with a charge-coupled device (CCD) camera (DMi8, Leica, Wetzlar, Germany, and Retiga 2000R, QImaging, Surrey, BC, Canada). After the confirmation of cell attachment and starting neurite growth, the inlet/outlet of the Cell Channel were replaced with 5 µL of fresh medium. The outlet and the inlet of the Test Substance Channel were then replaced with 4.5 µL of medium, and 4.5 µL of medium with 50 ng/mL NGF, respectively, to generate a gradient of NGF.

## 3. Results and Discussion

### 3.1. Size of the Microfluidic Channels

**Figure 3:**
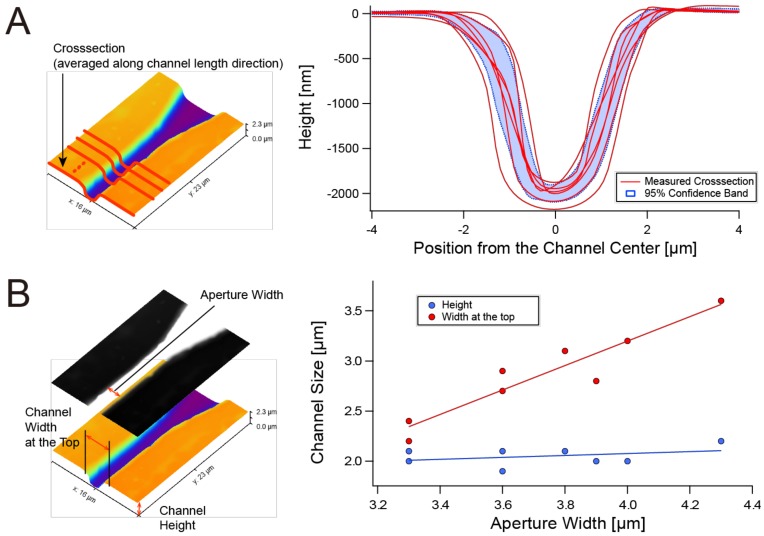
The size of PDMS channel features transferred from SU-8 photoresist patterned with diffused 185 nm/254 nm UV from the backside. (**A**) Variation in cross-sectional profiles of PDMS features cast from seven different SU-8 molds fabricated with the same photolithographic conditions. The microchannel profiles were obtained using atomic force microscopy (AFM). Each curve is an average of the crosssections over the full length of each thin channel. (**B**) Relationship between the size (width, height) of thin channels and the width of apertures that defines the pattern of the SU-8 layer. The microchannel size and the aperture width were obtained using AFM and laser confocal microscopy.

We evaluated the shape, stability and controllability of the cross-sectional size of the thin channel casted from SU-8 positive features patterned by 185 nm/256 nm-diffused UV backside exposure. Figure 3A shows the variability in the cross-sectional size of thin PDMS microchannel features cast from seven different SU-8 microchannel features. All of the channels had smooth bell-shaped cross-sections with the height of around 2 µm, allowing smooth priming for their cross-sectional size. Although no intentional changes in process parameters were made, there were distinct variations in the channel width. The average channel width with 95% confidence band (95% CB) was 3.61 ± 1.27 µm at a depth of 0.1 *h* from the top surface (*h*: channel height), and 2.14 ± 0.89 µm at the half height (0.5 *h*), whereas the average height with 95% CB was 2.00 ± 0.19 µm.

The profiles shown in Figure 3A were taken from separate SU-8 features that were patterned on the same substrate using the same photomask pattern, and thus they share the same parameters including SU-8 thickness, SU-8 exposure time, and PDMS curing temperature and time. Therefore, the variation in widths of Cr apertures under the SU-8 layer (see Figure 2) may contribute to the variation of the channel width. Figure 3B shows the relationship between the channel width and the aperture size. As expected, the channel width directly correlated to the aperture width. However, variation from the proportional relationship between aperture and channel widths was observed. Figure 3B also shows that the channel height is around 2 µm regardless of varying aperture width. The variation from the proportional relationship was within 100 nm.

The relationship between the channel width and the Cr aperture width shown in Figure 3B suggests that the variation in the Cr aperture width was directly reflected in the channel width. The use of a low-cost emulsion glass mask and a spot UV light source to expose positive photoresist for patterning the Cr aperture might affect the variation of the aperture width. The variation can also be minimized by adopting more expensive but still average-level photolithography equipment. Also, the thin channel was curved and determined by diffused UV. Therefore, the border surface of crosslinked and uncrosslinked SU-8 resin was not consistent compared to the straight sidewall feature defined by collimated light. Such variation may be decreased by controlling the solid content of SU-8, local temperature at baking, and the flow of fresh solvent over SU-8 during development.

However, the variations in channel size are still allowable for prototyping uses, because the fully crosslinked bell-shaped SU-8 structures are durable enough for multiple (50 times or more) silicone casting. A user can fabricate sufficient numbers of microfluidic chips even if only a few molds are usable.

The experimental results also confirmed the effectiveness of 185 nm UV for patterning the small features of 2 µm-height. Generally, coating a 2 µm-thick layer of SU-8 with high solid content, which ensures the successful development of small features, is not an easy task due to high viscosity and a rise in viscosity caused by solvent evaporation. Limited penetration depth of 185 nm UV enabled the generation of 2 µm-height features from a 10 µm-thick SU-8 layer. We found that the wavelength of 185 nm is the most suitable for microchannel patterning. Exposure using a germicidal lamp (256 nm only) failed due to swelling caused by insufficient crosslinking; exposure to a 172 nm excimer lamp did not generate features thicker than 200 nm (data not shown). The 185 nm UV was effective at fully crosslinking SU-8 photoresist and completing the development of around 2 µm-high features at low cost.

### 3.2. Gradient Generation

**Figure 4:**
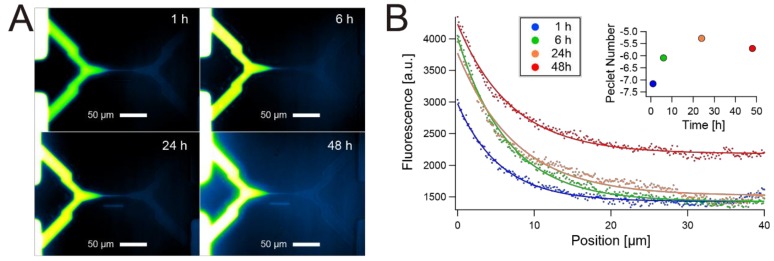
Concentration gradients generated on the H-shaped microfluidic configuration with the thin channel at the center. (**A**) Microscope image of fluorescence from Alexa Fluor 488 initially introduced to the bottom left end of the thin channel. Top right and bottom right ends of the thin channel were Laplace pressurized at the inlets/outlets after introducing the fluorophore. (**B**) Time evolution of fluorescence intensity profile along the thin channel region obtained from (A). Solid line curves represent fitting of intensity data points to Equation (2). Peclet numbers obtained from the fitting were also shown.

We were able to maintain the concentration gradient of test substances between the ends of the thin microchannel for days. Figure 4A shows an example of the concentration gradients of fluorescent dye (Alexa Fluor 488) after the dye solution was added to the Test Substances Channel inlet and outlet (Figure 1C). By making a slight backflow condition within an H-shaped channel (i.e., the inlet/outlet of Cell Channel were pressurized more than that of Test Substance Channel), the fluorophore intensity profile was persistent in the thin channel over a long period of time. We did not observe significant changes in gradients in the order of 10 minutes, and as shown in Figure 4, only a small change in gradient is observed between 6 h and 24 h. Figure 4B shows that a distinct difference in the fluorescent dye concentration was maintained in the thin channel for at least 48 h. If there was no pressure difference between the Test Substance Channel and the Cell Channel, the fluorophore diffused much more rapidly, and the gradient disappeared quickly.

The conventional model of one-dimensional diffusion from a constant point source, Equation (3), explains the rapid disappearance of the concentration gradient within pure diffusion conditions [18,19].

(3) $$C\left(x,t\right)=\left(C_{\max}-C_{\min}\right)\mathrm{erfc}\left(\frac{x}{2\sqrt{D\left(1+\kappa {\mathrm{Pe}}_{w}^{2}\right)t}}\right)+C_{\min}$$

where D is the molecular diffusion coefficient of the fluorophore (435 µm^2^/s for Alexa Fluor 488 [20]), ${\mathrm{Pe}}_{w}$ Peclet number is defined using the width as the relevant length scale, κ is the sidewall factor and is ≈0.0035 for the parabolic cross-section [19]. When we define the concentration at the diffusion front as $0.9\left(C_{\max}-C_{\min}\right)$, and even if we ignore the effect of dispersion in the channel width direction (i.e., ${\mathrm{Pe}}_{w}=0$), the predicted value of the diffusion front transit time was only 116.5 s for the 40 µm length. More ${\mathrm{Pe}}_{w}$ gives a shorter transit time. 

Although thinning of the microchannel decreases ${\mathrm{Pe}}_{w}$, it is not sufficient to extend the transit time to the order of days. Applying backpressure is necessary for the long-term maintenance of the concentration gradient. Although we did not directly observe the backflow under a microscope, the negative values of Peclet numbers calculated with Equation (3) (Figure 4B) indicate the existence of backflow. While Equation (3) assumes static conditions and constant concentration at the point source, it fitted well to the actual gradients. The thin channel may contribute to this good fit because the large flow resistance of the thin channel slows down the backflow rate close to the static condition, and the effect of small inflow on the source concentration is negligible.

Our H-shaped gradient generator with a thin channel has appropriate flow resistance and satisfies both the rapid introduction of test substance and long-term maintenance of gradients simultaneously. While maintaining the gradient for two days would be sufficient to test the effect of gradient on cells such as chemotaxis, the user can extend or shorten the diffusion in the thin channel by adjusting the pressure difference between two channels at the ends of the thin channel.

### 3.3. Guided Axon Elongation

Using a long-lasting concentration gradient as shown in Figure 4, we examined the axon elongation of primary DRG neurons depending on a gradient of nerve growth factor (NGF). NGF is a neurotrophic factor that contributes to axon guidance [21]. Therefore, axons from the DRG neurons would elongate towards the NGF source guided by the gradient that should be present at the thin channel.

**Figure 5:**
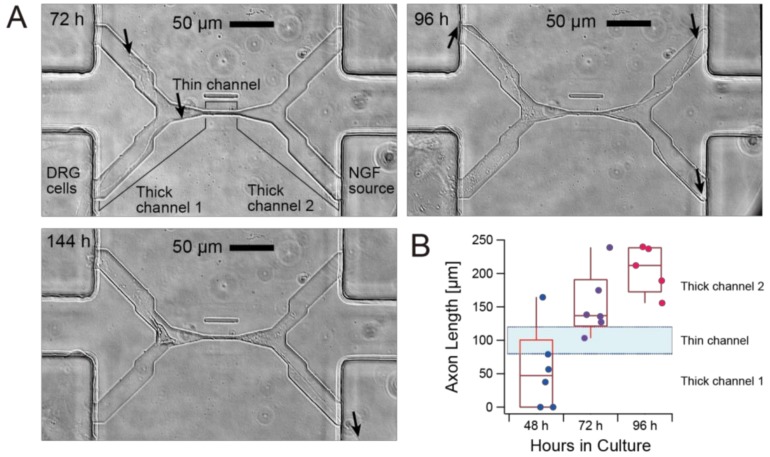
Axon elongation of primary nerve (dorsal root ganglion: DRG) cells from chick embryo in culture on the microfluidic chip. (**A**) Phase contrast images of axon elongation from the inlet of the cell channel (**bottom left**), the thick channel 1, the thin channel, the thick channel 2, and the source of nerve growth factor (NGF) (**bottom right**). Arrows denote the tip of an outgrowth axon. (**B**) Time evolution of the length of axons cultured on six different chips from the same mold. Dots above the “Thin Channel” band indicate that the axons passed through the thin channel, meaning that the axon is chemically separated from the cell body.

Figure 5 shows that a sharp gradient of NGF was sufficient to define the direction of axon elongation and was maintained for a long period because of minimal diffusion in the thin channel. Phase contrast microscopy was used to identity axons and cell bodies without disturbing the existing concentration gradient of NGF. As shown in Figure 5A, initially an axon tended to elongate along channel edges, and at 96 h the tips of axons reached all three other ends of the H-shaped channel. However, at 144 h, only the axon grown towards the NGF source selectively survived. This example shows that the direction and/or survival of axons are both attributed to NGF. Axon elongation to the NGF source was consistently observed when DRG cells were cultured in an NGF gradient. Figure 5B shows that the axon passed the thin channel within 72 h, and elongated toward the NGF source at least until 96 h for most cases.

As shown in Figure 5A, we could easily locate cell bodies and axons with conventional phase contrast microscopy without staining that would compromise cell viability. The H-shaped microfluidic configuration with a single axon separation channel also more easily enables the tracking of channel also elongation and directional responses of single axons compared with a prevailing microfluidic device that has multiple axon separation channels at the center of the H-shaped configuration [22]. Currently, it is difficult to track an axon back to the originating cell body due to the need to increase the cell density to maintain cell viability. Combining an appropriate coculture system with this H-shaped channel may enable single DRG cell survival and eventually single axon separation on chip. 

## 4. Conclusions

We have developed a 2 µm-height bell-shaped microfluidic channel that was replicated from a SU-8 photoresist mold defined by a 2 µm-wide line mask pattern. Such thin and smooth SU-8 features were successfully crosslinked with backside photolithography using diffused 185 nm UV light that can pattern small features of SU-8 that cannot be successfully crosslinked using UV of longer wavelengths. The average height and width at the half height of the channel, defined by a 2 µm-wide line mask pattern, were 2.00 ± 0.19 µm, and 2.14 ± 0.89 µm, respectively. In this technique, the only process that is unusual for conventional photolithography/soft lithography was a low-cost ozone lamp and sputter coating of Cr on a fused silica wafer. The Cr coating, however, can be replaced by purchasing a mask blank.

We have successfully demonstrated the generation of concentration gradients lasting for at least 48 h, taking advantage of the high fluid resistance of the thin channel and appropriate backpressure controlled by the amount of liquid at the end ports of the H-shaped channel. We have also demonstrated the directional elongation of chick embryo DRG neurons following gradients of NGF during cultivation for 96 h.

The fabrication of both thin SU-8 features and the gradient generation chip was simple enough to be implemented by non-experts. The gradient generator chip could provide opportunities for a wide range of experiments investigating single axon or other single cell behaviors in response to microscale concentration gradients.
